# Hypoxia-induced autophagy in triple negative breast cancer: association with prognostic variables, patients’ survival and response to neoadjuvant chemotherapy

**DOI:** 10.1007/s00428-023-03527-4

**Published:** 2023-03-20

**Authors:** Dina M. El-Guindy, Fatma MKh Ibrahim, Dina A. Ali, Hemat El-Sayed El-Horany, Nesreen M. Sabry, Rasha A. Elkholy, Wael Mansour, Duaa S. Helal

**Affiliations:** 1 Pathology Department, Faculty of Medicine, Tanta University, Tanta, Egypt; 2 Clinical Pathology Department, Faculty of Medicine, Tanta University, Tanta, Egypt; 3 Medical Biochemistry Department, Faculty of Medicine, Tanta University, Tanta, Egypt; 4 Clinical Oncology Department, Faculty of Medicine, Tanta University, Tanta, Egypt

**Keywords:** Hypoxia-inducible factor-1α (HIF-1α), miR-210, Beclin-1, Bcl-2, Triple-negative breast cancer (TNBC)

## Abstract

Autophagy is a cellular response to diverse stresses within tumor microenvironment (TME) such as hypoxia. It enhances cell survival and triggers resistance to therapy. This study investigated the prognostic importance of HIF-1α and miR-210 in triple negative breast cancer (TNBC). Also, we studied the relation between beclin-1 and Bcl-2 and their prognostic relevance in triple negative breast cancer. Furthermore, the involvement of hypoxia-related markers, beclin-1 and Bcl-2 in mediating resistance to neoadjuvant chemotherapy (NACT) in TNBC was evaluated. Immunohistochemistry was performed to evaluate HIF-1α, beclin-1 and Bcl-2 expression whereas, miR-210 mRNA was detected by quantitative reverse transcription PCR (q-PCR) in 60 TNBC patients. High HIF-1α expression was related to larger tumors, grade III cases, positive lymphovascular invasion, advanced stage, high Ki-67 and poor overall survival (OS). High miR-210 and negative Bcl-2 expression were related to nodal metastasis, advanced stage and poor OS. High beclin-1 was associated with grade III, nodal metastasis, advanced stage and poor OS. Also, high beclin-1 and negative Bcl-2 were significantly associated with high HIF-1α and high miR-210. High HIF- 1α, miR-210 and beclin-1 as well as negative Bcl-2 were inversely related to pathologic complete response following NACT. High beclin-1 and lack of Bcl-2 are significantly related to hypoxic TME in TNBC. High HIF-1α, miR-210, and beclin-1 expression together with lack of Bcl-2 are significantly associated with poor prognosis as well as poor response to NACT. HIF-1α and miR-210 could accurately predict response to NACT in TNBC.

## Introduction

Triple-negative breast cancer (TNBC) assigns to a subgroup of breast cancer (BC) determined by the absence of estrogen receptor (ER), progesterone receptor (PR), and human epidermal growth factor receptor 2 (HER2). TNBC phenotype is characterized by high heterogeneity, aggressive clinical behaviour and lack of treatment modalities [1]. Chemotherapy is considered the approved option for TNBC treatment; however, patients repeatedly acquire resistance. It is now widely accepted that the expression of tumour microenvironment (TME)-related factors could assist substantially in chemoresistance [2].

Tumour hypoxia, which is the lack of oxygen within a tumour, is one of the most common characteristics of the TME due to rapid cell growth and oxygen consumption. Hypoxia-inducible factor (HIF-1α), a nuclear transcription factor, is a marker of the hypoxic TME [3]. At present, it is well established that hypoxia serves as an independent unfavourable prognostic factor in several tumours and leads to the ultimate failure of most anticancer therapeutics [4, 5]. MicroRNAs (miRNAs) are classified as small non-coding, single-stranded RNAs (ribonucleic acids) which exerts essential roles in different fundamental processes through their influence on gene expression [6]. Recently, several miRNAs induced during hypoxia have been recognized and termed hypoxia-induced miRNAs or hypoxamiRs. Of them, miRNA-210 (miR-210) is the master hypoxamiR [7]. Hypoxia has been proved to enhance autophagy in diverse cellular conditions, and autophagy might behave as a survival mean for hypoxic cells by recycling their cellular contents [8].

Autophagy is a new focus of tumour research. Autophagy has a dual role; it is a barrier to hinder tumor invasion and supress tumorigenesis, and it is an adaptive reaction to the relatively harsh TME in encouraging tumor progression [9]. Beclin-1, the firstly recognized mammalian autophagy protein, is considered as a peculiar Bcl-2-interacting protein. The interplay between beclin-1 and Bcl-2 was described as an important regulator of autophagy and apoptosis. Disengaging beclin-1 from Bcl-2 triggers autophagy. Beclin-1 performs a vital role in the initiation and maturation of the autophagosome. Beclin-1 plays an essential role in different biological cellular processes, such as response to stress, aging, and cell death [10].

Autophagy levels were described to correlate with HIF-1α expression and have been related to early invasion in colonic carcinoma [11]. Furthermore, autophagy was demonstrated to exert an important role in promoting chemoresistance in lung cancer via hypoxia-related pathway [12].

Herein, this study aimed to investigate HIF-1α expression and miR-210 mRNA relative expression and their clinical relevance in TNBC. Moreover, we analysed the relationship between beclin-1 and Bcl-2 and their prognostic importance in TNBC. This study was extended to evaluate the involvement of hypoxia-related markers, beclin-1 and Bcl-2 in mediating neoadjuvant chemoresistance in TNBC.

## Patients and methods

### Study design and case selection

This is a prospective study performed at Pathology, Clinical Pathology, Medical Biochemistry and Clinical Oncology Departments, Faculty of Medicine, Tanta University during the period from June 2019 to June 2022. Sixty patients with TNBC were treated with neoadjuvant chemotherapy (NACT). All patients provided written, informed consent and approval of the institutional research ethics committee was obtained (Approval code: 35521/6521/6).

#### Eligibility criteria

Eligibility criteria included TNBC cases with no evidence of distant metastasis at the beginning of the study, Karnofsky performance status ≥ 70, adequate bone marrow reserve and good renal function (creatinine clearance ≥ 60 mL/min), whereas those who developed metastatic disease, had other malignancies or non-malignant systemic disease as well as patients ineligible for neoadjuvant chemotherapy were excluded.

### Clinical assessment

All cases underwent full laboratory investigations to figure out their tolerability to NACT. Diagnosis was established through core biopsy obtained before starting neoadjuvant treatment. Clinical staging work-up included computerised tomography (CT) chest, abdomen and pelvis, along with bone scan. TNM staging was applied according to the American Joint Committee on Cancer (AJCC) [13].

### Histopathologic evaluation

TNBC cases were determined following the American Society of Clinical Oncology/College of American Pathologists (ASCO/CAP) guidelines [14] as ER and PR negative (nuclear staining in < 1% of tumour cells) and lack HER2 overexpression (no staining, or weak incomplete membrane staining in less than 10% of tumour cells by immunohistochemistry) or oncogene amplification [Dual-probe HER2/CEP17 < 2.0 with an average HER2 copy number < 4.0 signals/cell by fluorescent in situ hybridization (FISH)]. Haematoxylin and eosin-stained sections were examined for the histologic type, tumour grade and lymphovascular invasion (LVI). Nottingham grading system was adopted for assessment of tumor grade [15, 16].

### Immunohistochemical staining

Immunohistochemical staining was performed in Dako Autostainer Link 48 on sections obtained from pre-NACT core biopsy specimens using HIF-1α rabbit monoclonal antibody (EP1215Y, Biocare Medical, Concord, CA, USA), beclin-1 mouse monoclonal antibody (5A11, Cat#: ABM40317, Wuhan, China) and Bcl-2 mouse monoclonal antibody (CA IR614; Dako: Agilent Dako Company, USA). Deparaffinization and antigen retrieval through Dako PT Link unit along with treatment with peroxidase blocking reagent for 5 min were done before incubation with the primary antibodies for 30 min. Subsequently, slides were incubated with horseradish peroxidase polymer reagent for 20 min and diaminobenzidine chromogen for 10 min. Slides were then counterstained with haematoxylin.

#### Assessment of immunostaining

HIF-1α exhibited mixed subcellular localization both in the nucleus and the cytoplasm of malignant cells. Scoring was performed based on the intensity and the extent of immunostaining as follows: complete lack of staining, weak cytoplasmic staining regardless of the extent, strong cytoplasmic staining in < 50% of tumor cells and/or nuclear staining in < 10% of tumor cells were considered low expression, whereas strong cytoplasmic staining in > 50% of tumor cells and/or nuclear staining in > 10% of tumor cells were regarded as high expression [17]. Beclin-1 and Bcl-2 were detected as brownish cytoplasmic staining. For beclin-1, both the intensity and the percentage of positive tumor cells were evaluated. Intensity was graded as follows: 1, light yellow; 2, brownish yellow; 3, brownish, whereas the percentage of positive cells was scored as follows: 1, ≤ 10% positive cells; 2, 11–50%; 3, 51–75% positive cells; 4, > 75% positive cells. The final score was obtained by multiplying the intensity and the percentage of positivity scores. Score ≤ 3 was classified as the low expression group; where score > 3 was classified as high expression group [18]. Bcl-2 expression was analysed using the percentage of positively stained cells. Cytoplasmic staining in more than 10% of tumour cells was regarded as positive [19].

### Detection of miR-210 mRNA Gene Expression by Quantitative Reverse-Transcription PCR

Breast core biopsies were obtained from all patients before receiving any treatments and immediately stored at − 80 °C for molecular study. Total RNA was extracted using miRNeasy Mini kits (Cat. no. 217061; Qiagen, Hilden, Germany) according to the manufacturer’s instructions. The concentration of the extracted RNA was measured using a NanoDrop® 1000 spectrophotometer (Thermo Scientific, Wilmington, DE, USA) at 260 nm while the purity was checked using absorbance ratio at 260/280 nm. The RNA was reversely transcribed using a MiScript® II RT Kit (Cat. no. 218161; Qiagen, Germany) according to the manufacturer’s protocol on a Biometra thermal cycler (Biometra GmbH, Gttingen, Germany), then stored at − 20 °C for the subsequent PCR step. The miR-210 mRNA expression was quantified using a miScript SYBR® Green PCR Kit (Cat. no. 218073; Qiagen, Germany) according to the manufacturer’s guide and the endogenous control, RNU6B, was used for normalization. The reaction was performed on the Step One q-PCR system (Applied Biosystems, CA, USA) with the following thermal profile: Hold at 95 °C for 20 s, then 40 cycles (denaturation at 95 °C for 15 s and annealing/extension at 60 °C for 60 s). The primer sequence of miR-210 was as follows: forward: 5′-CUGUGCGUGUGACAGCGGCUGA-3′and reverse: 5′-AGCCGCUGUCACACGCACAGUU-3′and for the reference gene (RNU6B): forward: 5′- AAAATTGGAACGATACAGAGA -3′ and reverse: 5′- AAATATGGAACGCTTCACGAA -3′. The miR-210 relative expression was quantified on the basis of the cycle threshold (CT) and normalized with RNU6B expression with the formula 2 − ΔCT where (ΔCt = Ct (miR-210)—Ct (RNU6B)) [20].

### Treatment protocol and evaluation of the pathologic response

All patients received neoadjuvant chemotherapy in the form of paclitaxel and carboplatin [paclitaxol 80 mg/m^2^ at day 1,8,15 and carboplatin; area under curve (AUC)] 5 day 1 repeated every 21 days, for 4–6 cycles). Adequate bone marrow, liver and renal functions were evaluated one day before each NACT cycle. Mastectomy was done after completion of NACT therapy. The follow-up program consisted of clinical and radiological assessment every 3 months for 2 years.

Pathologic tumour response was evaluated in all cases according to AJCC [21]. Pathologic complete response (pCR) was identified as complete absence of all invasive tumour cells from breast tissue and regional lymph nodes.

Patients achieved pCR following surgery and completed their neoadjuvant cycles underwent follow up. On the other hand, patients who did not achieved pCR received post operative adjuvant chemotherapy in the form of capecitabine (1000- 1250 mg/m^2^ per oral twice daily every 14 days repeated every 21 days for 6 cycles).

All patients received post operative radiotherapy (RTH), either chest wall irradiation or whole breast irradiation. As regards chest wall irradiation; the target includes ipsilateral chest wall, the mastectomy scar and the drain if possible. The RTH dose is 45–50 GY in 25 to 28 fractions, with or without scar boost 1.8- 2 GR per fraction to a total dose of approximately 60–66 GY, 5 days per week. Chest wall scar boost may be delivered with or without bolus, by either electron or photon. In whole breast irradiation; the target is the breast tissue at risk. The RTH dose is either hypofractionated dose of 40–42.5 Gy or conventional fractionation total dose,45–50 GY, in 25–28 fractions, daily 5 days per week, boost to the tumor bed in high risk of recurrence, dose 10–16 GY in 4 to 8 fractions.

Regional nodal irradiation was applied for the patients with high risk for nodal recurrence, RTH dose 45–50 GY, in 25–28 fractions, daily 5 days per week.

### Statistical analysis

SPSS (Statistical Package for the Social Sciences, version 23.0) was applied to analyse data. Qualitative data were expressed as frequencies whereas quantitative data were expressed as mean ± SD. Normality was verified using Shapiro–Wilk test. Difference between groups was tested using Chi -square, Fisher’s exact and Monte Carlo tests. Difference between means of groups was carried out using Student t-test for normally distributed variables and Mann–Whitney for non-normally distributed variables. OS was calculated from the date of diagnosis to the date of death, or the last follow-up (2 years in this study). Kaplan–Meier curves were plotted, and the log rank test was used for comparison between groups. Cox proportion hazards regression model was applied to evaluate the significance of various prognostic factors on patients’ survival. To analyse the predictive power of the significant parameters for pCR, the receiver operating characteristics (ROC) curve was performed and area under the curve (AUC) value was determined. An AUC value of > 0.7 was considered sufficient for accurate prediction [22]. *P* values of < 0.05 were considered statistically significant.

## Results

### Clinicopathologic characteristics of the studied cases

**Fig. 1:**
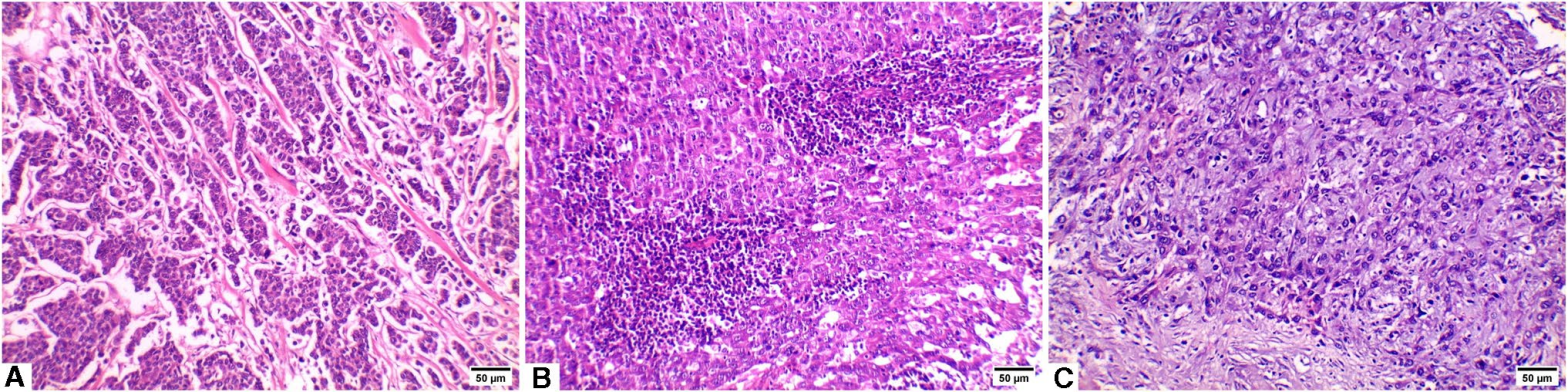
Hematoxylin and eosin (H and E) stained sections of TNBC cases (× 200). **A)** Invasive breast carcinoma – no special type (IBC-NST), **B)** IBC with medullary pattern, **C)** Metaplastic carcinoma

This study included 60 cases with TNBC. Table 1 demonstrates the clinicopathologic characteristics of the studied TNBC cases. The mean age of the TNBC cases was 47.48 ± 8.68 years. Tumor size had a mean of 4.33 ± 1.22 cm. The majority of cases were invasive breast carcinoma of no special type (IBC-NST) [53 cases (88.33%)]. Grade III cases constituted 36 cases (60%) whereas stage III was detected in 33 cases (55%). Forty-seven cases (78.3%) were positive for nodal metastasis and 33 cases (55%) displayed lymphovascular invasion. Ki-67 proliferation index was > 14% in 46 cases (76.7%). By the end of follow up time, 15 cases (25%) have died. Representative images of H and E-stained sections of the studied TNBC cases are included in Fig. 1.

**Table 1:** Clinicopathologic characteristics

	Total N (%)
Age (years)	
mean ± SD	47.48 ± 8.68
Range	32–66
Menopausal status	
Premenopausal	34 (56.7)
Postmenopausal	26 (34.3)
Size (cm)	
Mean ± SD	4.33 ± 1.22
Range	1.8–5.9
Histologic type	
Invasive breast carcinoma—NST	53 (88.33)
IBC with medullary pattern	4 (6.67)
Metaplastic carcinoma	3 (5)
Pathologic grade	
II	24 (40)
III	36 (60)
Lymphovascular invasion	
No	27 (45)
Yes	33 (55)
Nodal metastasis	
Negative	13 (21.7)
Positive	47 (78.3)
Clinical stage	
II	27 (45)
III	33 (55)
Ki-67	
≤ 14%	14 (23.3)
> 14%	46 (76.7)
Adjuvant Chemotherapy	
No	39 (65)
Yes	21 (35)
Pathologic response	
Pathologic Complete response (pCR)	39 (65)
Non-pCR	21 (35)
Death	
Positive	15 (25)
Negative	45 (75)

*IBC*: invasive breast carcinoma, *NST*: no special type, *pCR*: pathologic complete response

### HIF-1α and miR-210 in relation to clinicopathologic parameters

**Fig. 2:**
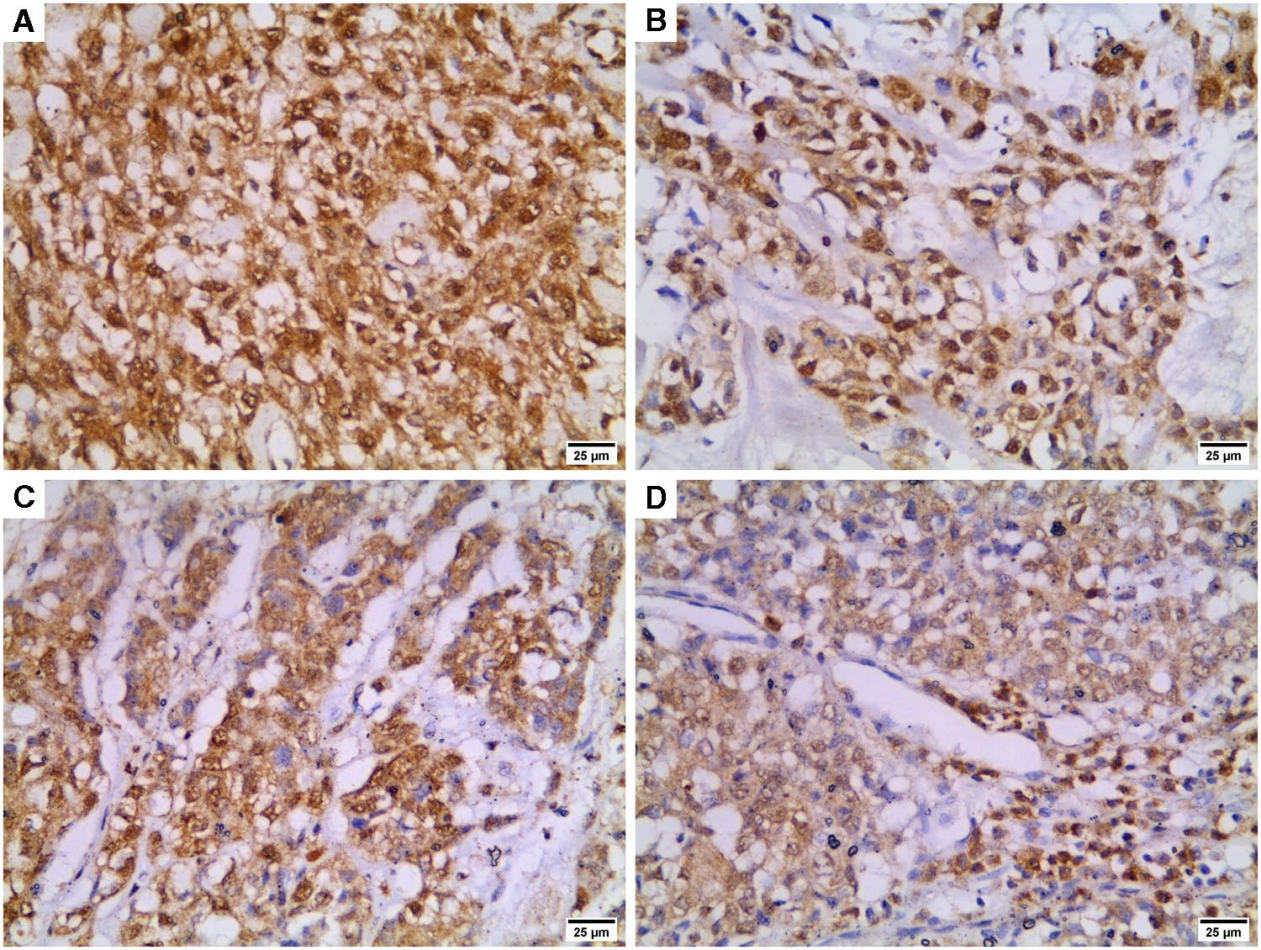
Representative images demonstrating HIF-1α immunohistochemical expression in TNBC cases (× 400). **A)** Strong cytoplasmic staining with nuclear expression, **B)** weak cytoplasmic staining with nuclear expression, **C)** strong cytoplasmic staining with some cells exhibiting nuclear staining, **D)** weak cytoplasmic staining with positive nuclear expression in some cells

Representative images of HIF-1α immunostaining are demonstrated in Fig. 2. High HIF-1α expression was detected in 28 (46.67%) cases. High HIF-1α expression was significantly associated with large tumor size, grade III cases, positive LVI, stage III cases and high Ki-67 expression (p = 0.027, 0.027, 0.017, 0.017, 0.001 respectively). Regarding miR-210 expression, high miR-210 expression was significantly associated with positive nodal metastasis (median 1.56, range 0.15–5.10), and stage III tumors (median 1.68, range 0.15–5.10) [p = 0.025 and 0.033 respectively]. Table 2 demonstrates the relation between HIF-1α and miR-210 and clinicopathologic characteristics of the studied cases.

**Table 2:** Hypoxia-related markers in relation to clinicopathologic parameters

	Total	HIF-1α expression			miR-210 relative expression	
		Low *N* = 32 (%)	High *N* = 28 (%)	*P* value	Median (Range)	*P* value
Age (years)						
mean ± SD		46.88 ± 8.32	48.18 ± 9.18	0.566	R = -0.054	0.681
Premenopausal	34	21 (61.8)	13 (38.2)	0.134	1.15 (0.10–5.10)	0.848
Postmenopausal	26	11 (42.3)	15 (57.7)		1.41 (0.15–3.1)	
Tumor size (cm)						
Mean ± SD		4.00 ± 1.34	4.69 ± 0.96	0.027*	R = 0.157	0.230
Histologic types						
Invasive breast carcinoma—NST	53	29 (54.7)	24 (45.3)	0.168	1.3 (0.10–5.10)	0.159
IBC with medullary pattern	4	3 (75)	1 (25)		0.95 (0.80–1.10)	
Metaplastic carcinoma	3	0 (0)	3 (100)		1.63 (0.60–3.10)	
Pathologic grade						
II	24	17 (70.8)	7 (29.2)	0.027*	1.05 (0.10–2.40)	0.154
III	36	15 (41.7)	21 (58.3)		1.64 (0.15–5.10)	
Lymphovascular invasion						
No	27	19 (70.4)	8 (29.6)	0.017*	1.28 (0.15–5.10)	0.275
Yes	33	13 (39.4)	20 (60.6)		1.5 (0.10–5.10)	
Nodal metastasis						
Negative	13	10 (76.9)	3 (23.1)	0.066	1.3 (0.10–2.40)	0.025*
Positive	47	22 (46.8)	25 (53.2)		1.56 (0.15–5.10)	
Clinical stage						
II	27	19 (70.4)	8 (29.6)	0.017*	1.1 (1.10–2.40)	0.033*
III	33	13 (39.4)	20 (60.6)		1.68 (0.15–5.10)	
Ki-67						
≤ 14%	14	13 (92.9)	1 (7.1)	0.001*	0.9 (0.15–3.20)	0.063
> 14%	46	19 (41.3)	27 (58.7)		1.54 (0.10–5.10)	

^*^significant (p < 0.05), *IBC*: invasive breast carcinoma, *NST*: no special type

### Beclin-1 and Bcl-2 in relation to clinicopathologic parameters

**Fig. 3:**
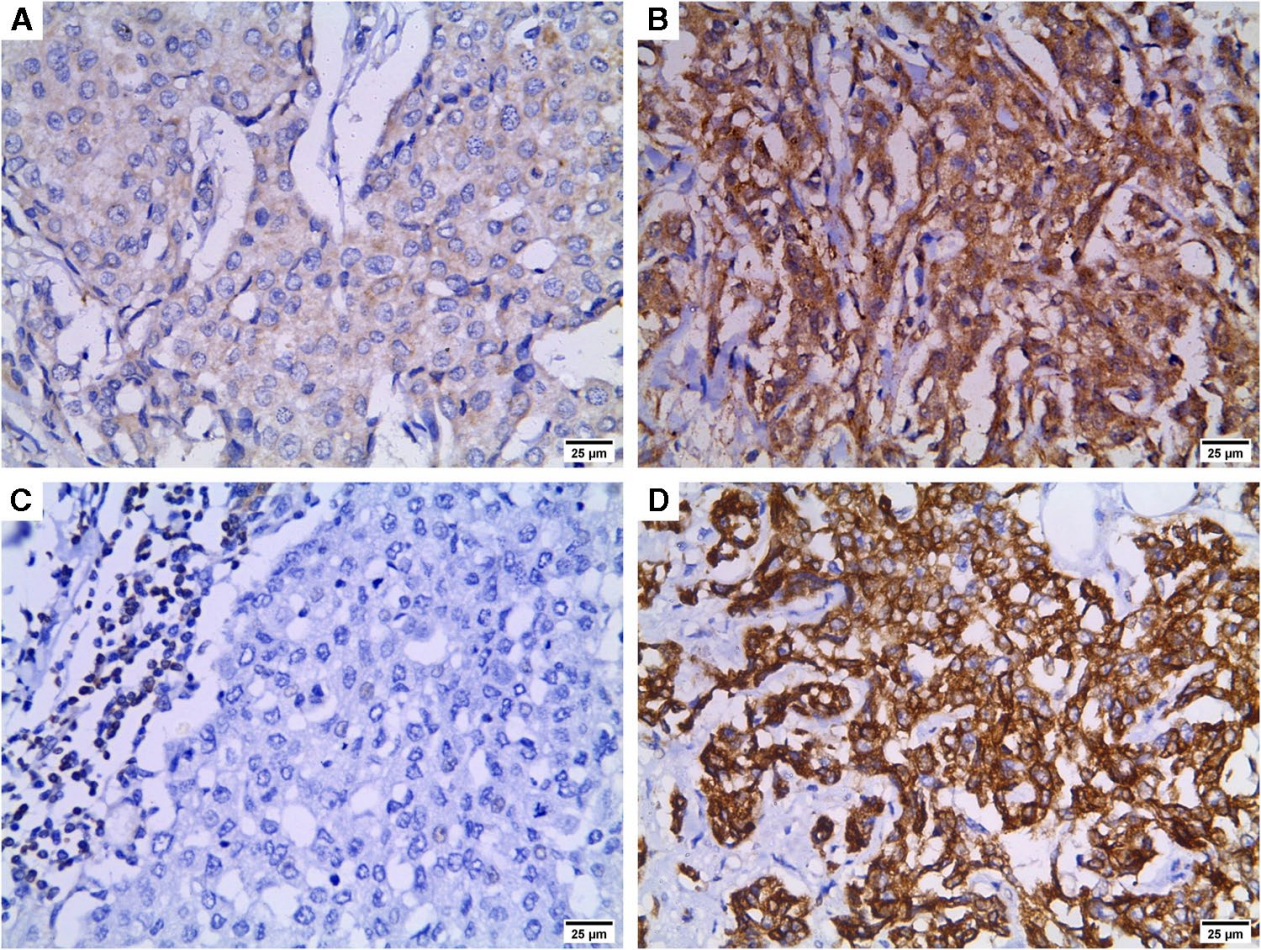
Beclin-1 and Bcl-2 immunostaining in TNBC cases (× 400). **A)** low beclin-1 expression, **B)** High beclin-1 immunostaining, **C)** Negative Bcl-2 expression in tumor cells with positive immunostaining of the infiltrating lymphocytes, **D)** positive Bcl-2 expression

Table 3 and Fig. 3 demonstrate beclin-1 and Bcl-2 results. Among the studied 60 TNBC case, 37 cases (61.67%) displayed high cytoplasmic beclin-1 immunostaining whereas positive Bcl-2 expression was detected in 25 cases (41.67%). Significant associations were detected between high beclin-1 expression and grade III cases, positive nodal metastasis and stage III cases (p = 0.039, 0.021 and 0.003 respectively). On the contrary, negative Bcl-2 expression was significantly associated with positive nodal metastasis and stage III cases (p = 0.030 and 0.018 respectively).

**Table 3:** Beclin-1 and Bcl-2 expression in relation to clinicopathologic parameters

	Total	Beclin-1 expression			Bcl-2 expression		
		Low *N* = 23 (%)	High *N* = 37 (%)	*P* value	Negative *N* = 35 (%)	Positive *N* = 25 (%)	*P* value
Age (years)							
mean ± SD		46.70 ± 7.14	47.97 ± 9.56	0.584	48.63 ± 8.99	45.88 ± 8.12	0.230
Menopausal status							
Premenopausal	34	14 (41.2)	20 (58.8)	0.604	17 (50)	17 (50)	0.134
Postmenopausal	26	9 (34.6)	17 (65.4)		18 (69.2)	8 (30.8)	
Tumor size (cm)							
Mean ± SD		4.36 ± 1.12	4.31 ± 1.30	0.866	4.28 ± 1.29	4.39 ± 1.14	0.730
Histologic types							
Invasive breast carcinoma—NST	53	20 (37.7)	33 (62.3)	0.131	31 (58.5)	22 (41.5)	0.138
IBC with medullary pattern	4	3 (75)	1 (25)		1 (25)	3 (75)	
Metaplastic carcinoma	3	0 (0)	3 (100)		3 (100)	0 (0)	
Pathologic grade							
II	24	13 (54.2)	11 (45.8)	0.039*	12 (50)	12 (50)	0.285
III	36	10 (27.8)	26 (72.2)		23 (63.9)	13 (36.1)	
Lymphovascular invasion							
No	27	10 (37)	17 (63)	0.852	16 (59.3)	11 (40.7)	0.895
Yes	33	13 (39.4)	20 (60.6)		19 (57.6)	14 (42.4)	
Nodal metastasis							
Negative	13	9 (69.2)	4 (30.8)	0.021*	4 (30.8)	9 (69.2)	0.030*
Positive	47	14 (29.8)	33 (70.2)		31 (66)	16 (34)	
Clinical stage							
II	27	16 (59.3)	11 (40.7)	0.003*	11 (40.7)	16 (59.3)	0.018*
III	33	7 (21.2)	26 (78.7)		24 (72.7)	9 (27.3)	
Ki-67							
≤ 14%	14	8 (57.1)	6 (42.9)	0.098	6 (42.9)	8 (57.1)	0.180
> 14%	46	15 (32.6)	31 (67.4)		29 (63)	17 (37)	

^*^significant (p < 0.05), *IBC*: invasive breast carcinoma, *NST*: no special type

### Beclin-1 and Bcl-2 in relation to hypoxia-related markers

**Fig. 4:**
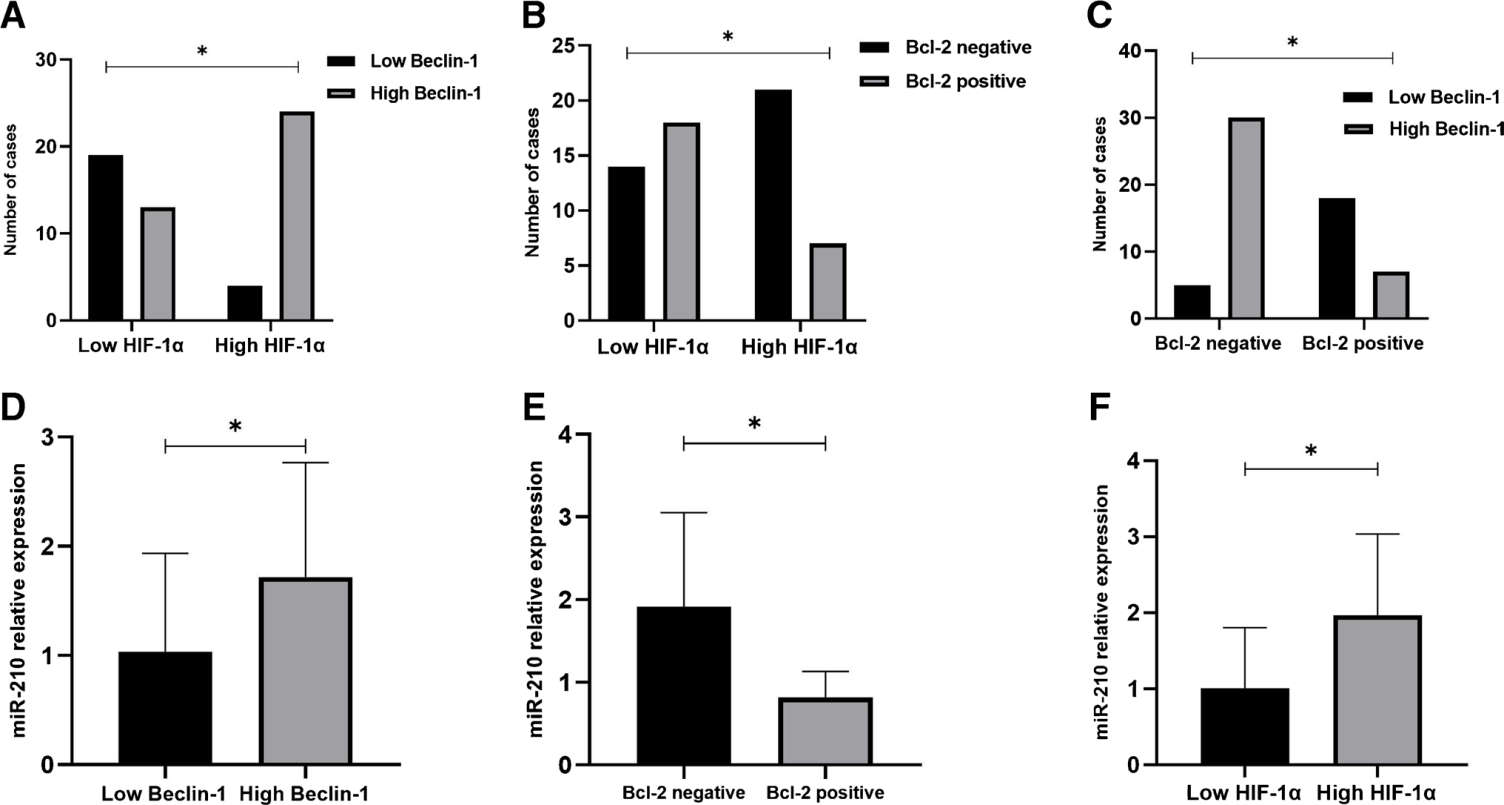
Beclin-1 and Bcl-2 expression in relation to hypoxia-related markers: significant associations were detected between **A)** HIF-1α and beclin-1 (p < 0.001; Fisher-exact test); **B)** HIF-1α and Bcl-2 (p = 0.014; Chi-square test); **C)** beclin-1 and Bcl-2 (p < 0.001; Chi-square test); **D)** beclin-1 and miR-210 (p < 0.001; Mann–Whitney test); **E)** Bcl-2 and miR-210 (p = 0.001; Mann–Whitney test); **F)** HIF-1α and miR-210 (p < 0.001; Mann–Whitney test)

Significant associations were detected between high beclin-1, negative Bcl-2 and hypoxic TME. High beclin-1 and negative Bcl-2 were significantly related to high HIF-1α (p < 0.001 and 0.014 respectively) and higher miR-210 expression (p < 0.001 and 0.001 respectively). Moreover, high HIF-1α expression was significantly related to high miR-210 expression levels (p < 0.001). In addition, the association between beclin-1 and Bcl-2 was significant as high beclin-1 expression was significantly associated with negative Bcl-2 (p < 0.001) as illustrated in Fig. 4.

### Hypoxia-related markers and autophagy in relation to patients’ survival

**Fig. 5:**
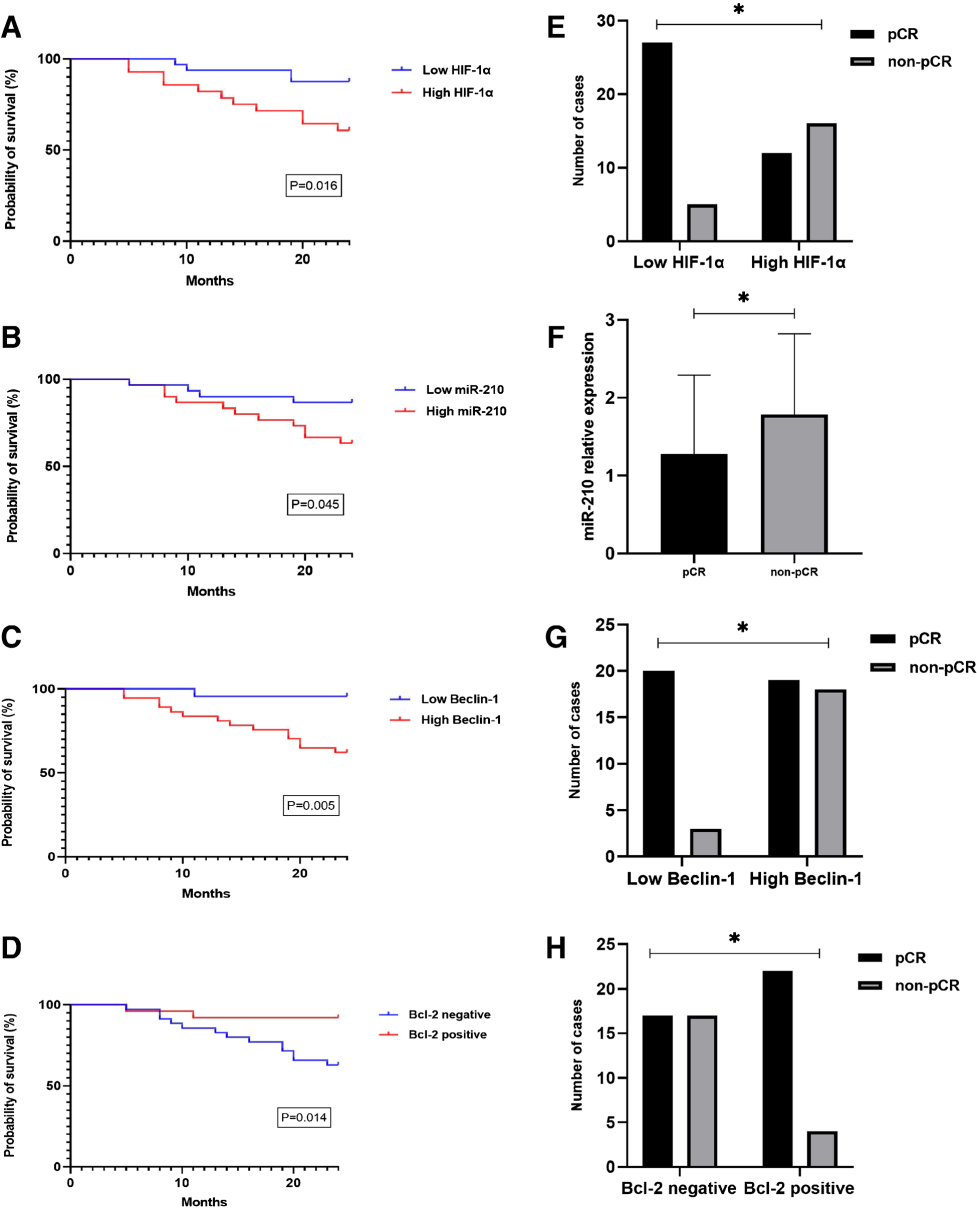
Hypoxia related markers (HIF-1α and miR-210), beclin-1 and Bcl-2 in relation OS and pathologic response to NACT in TNBC cases. **A)** HIF-1α and OS (significant, p = 0.016, Log-rank test); **B)** miR-210 and OS (significant, p = 0.045, Log-rank test, the median was used as a cut-off point to sort cases into low miR-210 and high miR-210 groups); **C)** beclin-1 and OS (significant, p = 0.005, Log-rank test); **D)** Bcl-2 and OS (significant, p = 0.014, Log-rank test); **E)** HIF-1α and pathologic response (significant, p < 0.001, Chi-square test); **F)** miR-210 and pathologic response (significant, p = 0.002, Mann–Whitney test); **G)** beclin-1 and pathologic response (significant, p = 0.005, Fisher-exact test); **H)** Bcl-2 and pathologic response (significant, p = 0.040, Chi-square test). OS: overall survival; NACT: neoadjuvant chemotherapy; TNBC: triple negative breast cancer

The OS rates for cases with high HIF-1α expression were significantly lower than those with low HIF-1α (Fig. 5A). The 1- and 2- year OS rates in the high HIF-1α group were 82% and 61% respectively compared to 94% and 88% respectively in the low HIF-1α group (p = 0.016). High miR-210 mRNA relative expression was significantly associated with poor OS compared to low expression (1- and 2- years OS rates were 90% and 87% in cases with low miR-210 levels versus 87% and 63% respectively in cases with high miR-210 levels) [Fig. 5B]. Furthermore, high beclin-1 expression was significantly associated with lower OS compared to low beclin-1 expression (1- and 2- years OS rates were 96% for both in cases with low beclin-1 versus 84% and 62% respectively in cases with high beclin-1) as demonstrated in Fig. 5C. As regards Bcl-2, there was a significant difference in OS between positive and negative groups (p = 0.014); the 1- and 2- OS rates were 92% for both in Bcl-2 positive group compared to only 86% and 63% in Bcl-2 negative group (Fig. 5D).

To further analyse the potential factors influencing the prognosis of the studied cases, the multivariate Cox regression model was performed. High HIF-1α and high beclin-1 expression were significantly associated with poor survival (HR, 7.561; 95% CI 1.210–47.243; p = 0.030 and HR, 30.009; 95% CI 1.941–463.878; p = 0.015 respectively) as illustrated in Table 4.

**Table 4:** Cox regression analysis of overall survival

Variables	Multivariate analysis	
	*P* value	HR (95% CI)
Pathologic grade	0.122	0.352 (0.238–1.562)
Nodal metastasis	0.070	0.197 (0.034–1.142)
HIF-1α	0.030*	7.561 (1.210–47.243)
Beclin-1	0.015*	30.009 (1.941–463.878)
Bcl-2	0.667	0.702 (0.139–3.531)
miR-210	0.303	0.610 (0.238–1.562)

^*^significant (p < 0.05), *HR*: hazard ratio, *CI*: confidence interval

### Hypoxia-related markers and autophagy in relation to NACT response

**Fig. 6:**
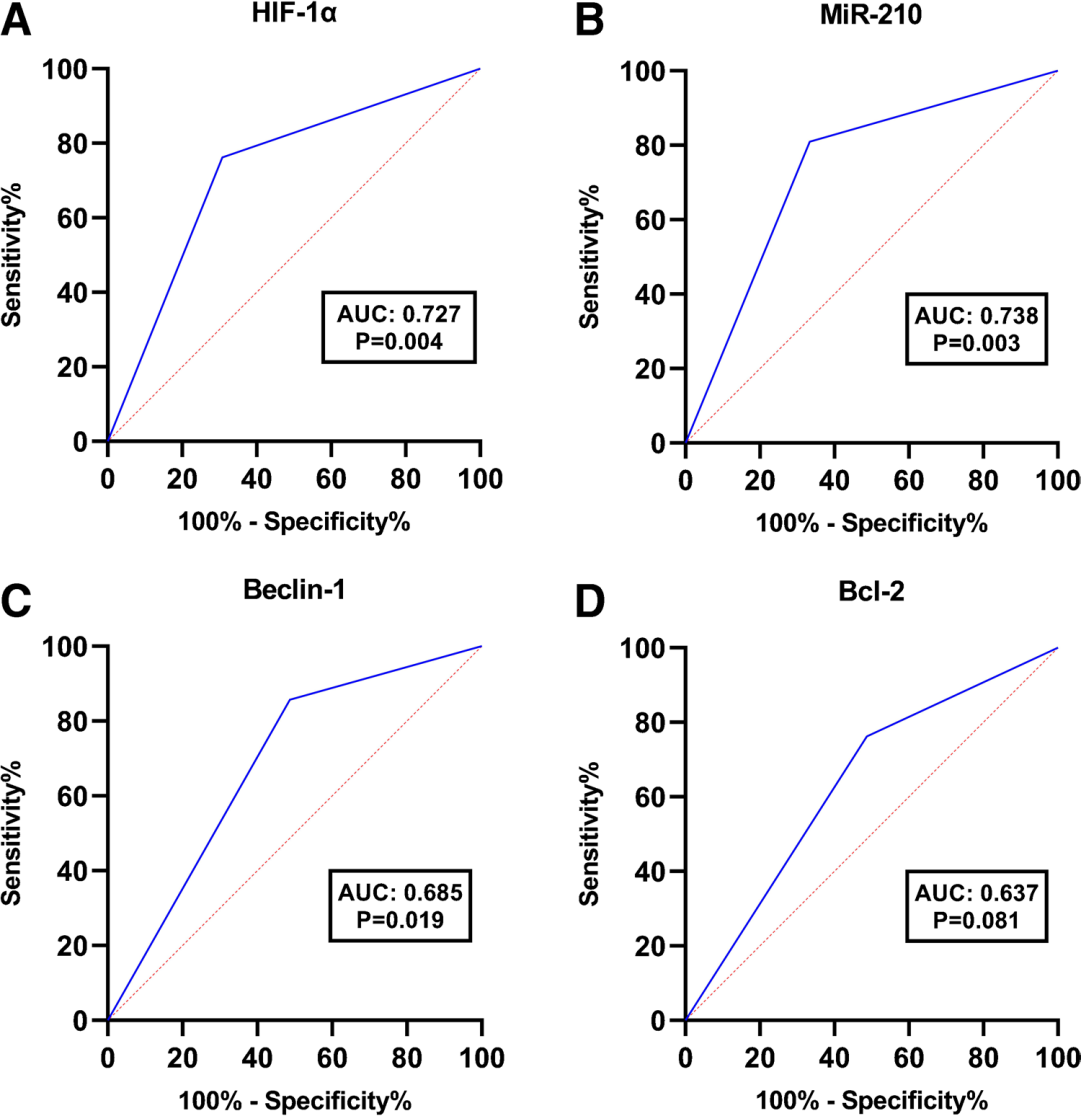
ROC curves for pathologic response prediction. **A)** HIF-1α**; B)** miR-210**; C)** beclin-1**; D)** Bcl-2. ROC; receiving operator curve

Figure 5 (E–H) demonstrates HIF-1α, miR-210, beclin-1 and Bcl2 expression in relation to post NACT pathologic response. Cases exhibiting hypoxic TME, identified by high HIF-1α and high miR-210 expression, were inversely associated with pCR. Moreover, high beclin-1 as well as lack of Bcl-2 expression were significantly associated with lower rate of pCR. The ROC curve was generated to analyse the predictive ability of the studied markers in differentiating pCR from non-pCR (Fig. 6). The accurate prediction was obtained by HIF-1α and miR-210 (AUC > 0.7). HIF-1α displayed AUC of 0.727, a sensitivity of 76.2% and a specificity of 69.2%. AUC in miR-210 was 0.738 with a sensitivity of 81% and a specificity of 66.7%. As regards beclin-1 and bcl-2, the AUC were 0.685 and 0.637 respectively.

## Discussion

Autophagy plays bipolar roles in cancer promotion and suppression. Stressful situations such as hypoxia as well as oxidative stress trigger autophagy to sustain the cellular homeostasis [23]. Recent studies throw light on autophagy due to its intimate relation with both tumor development and therapy resistance. This study was proposed to investigate the prognostic relevance of hypoxic TME in TNBC. Moreover, the association between beclin-1 and Bcl-2 in TNBC has been analysed. Also, the involvement of hypoxia-induced autophagy in mediating neoadjuvant chemoresistance in TNBC was evaluated.

In this study, HIF-1α immunohistochemical expression within tissue specimen as well as miR-210 mRNA relative expression were used to assess hypoxic TME within TNBC. High HIF-1α expression was detected in 28 cases (46.67%). Similar results were provided by Ong et al. [24], whereas Nalwoga et al. [25] and Yehia et al. [26] reported higher and lower HIF-1α frequencies respectively. Their analyses relied purely on the nuclear expression of HIF-1α and neglected the cytoplasmic staining. It is worth mentioning that, in this study, both nuclear and cytoplasmic localization were considered for HIF-1α evaluation. Supposing nuclear HIF-1α expression is indicative of its activity within the nucleus, HIF-1α protein is manufactured and degraded in the cytoplasm. Cytoplasmic expression, parallel to nuclear, is a precise feature and either cytoplasmic or nuclear localization is demonstrative of HIF-1α up-regulation [17].

Analysing the associations between hypoxic TME and tumor prognosis, the current work demonstrated significant associations between high HIF-1α expression and large tumor size, poorly differentiated and advanced stage cases, positive LVI and high Ki-67 proliferation index. In addition, high levels of miR-210 were significantly related to positive nodal metastasis and advanced cases. Furthermore, poor OS rates were reported with high HIF-1α expression and high miR-210 mRNA relative expression.

This was in accordance with Cui and Jiang [27] who concluded that HIF-1α expression was an independent factor predicting poor OS and that HIF-1α expression was significantly associated with older age, larger tumor, high grade, positive nodal involvement and TNM stage in TNBC. Similarly, Dales et al. [28] reported a significant relationship between HIF-1α and poor prognosis being related to poor clinicopathological variables as well as shorter patients’ survival in breast cancer. Furthermore, HIF-1α expression has been linked to poor prognosis in different cancers [29–31]. On the other hand, Ong et al. [24] reported that HIF-1α overexpression didn’t provide any associations with clinicopathologic characteristics in TNBC. Recently, Shamis et al. performed a systematic review and meta-analysis on 30 eligible studies (involving 6201 patients) on the prognostic value of HIF-1α in BC patients. They concluded that HIF-1α overexpression was significantly related to worse disease-free survival and OS. They have also demonstrated that the study population, antibodies used, and scoring methods applied were shown to influence HIF-1α expression [32]. HIF-1α is crucial regulator of cellular response to hypoxia as it affects most of “hallmarks” of cancer. HIF-1α plays important roles in tumorigenesis, metabolic reprogramming, angiogenesis, immune evasion. Furthermore, HIF-1α influences resistance to chemotherapy and radiation therapy via diverse mechanisms [33].

Along with our results, Toyama et al. [34] demonstrated significant associations between high miR-210 and poor prognostic parameters in TNBC. Additionally, Wu et al. [35] analysed miR-210 within breast cancer tissue using q-PCR, they described that miR-210 expression was significantly correlated with nodal metastasis, high grade and advanced stage. On the contrary, McCormick et al. [36] revealed an association between HIF-1α-induced miR-210 expression and favourable prognostic parameters in renal cell carcinoma. MiR-210 displays oncogenic features, as it is frequently increased in different carcinomas including breast, lung and pancreatic cancers [37]. Plentiful studies dealing with the diverse genes targeted by HIF-1α-induced miR-210 overexpression have displayed its vast involvement in tumor proliferation, apoptosis, angiogenesis, invasiveness, and treatment resistance [38].

The present study demonstrated a significant association between HIF-1α expression within TNBC and miR-210 mRNA relative expression as high HIF-1α expression was associated with high levels of miR-210. In a study by Li et al. [39], miR-210 overexpression, under hypoxic conditions, was positively correlated with increased HIF-1α expression in ovarian cancer. This enhanced cellular proliferation and tumor growth. Besides, another study by King et al. [40] suggested that miR-210 elevated following hypoxia could exert essential functions in enhancing tumour progression in response to hypoxia. MiR-210 serves as downstream of HIF-1α, which is frequently upregulated in different cancers under hypoxia.

The interplay between the antiapoptotic protein Bcl-2 and the autophagy protein beclin-1 has been demonstrated to coordinate the switch between the autophagy and apoptosis. Autophagy may stimulate or supress apoptosis based on diverse stresses within the TME [41]. The present study focused on assessing beclin-1 and Bcl-2 expression and their prognostic importance in TNBC. In the current work, beclin-1 displayed high expression in 37 cases (61.67%). High beclin-1 was significantly related to poorly differentiated cases, positive nodal metastasis as well as advanced stage cases. In addition, high beclin-1 expression was significantly related to poor OS.

Wang et al. [42] and Hamurcu et al. [43] studied beclin-1 expression in BC and reported that beclin-1 expression was highest in TNBC group. They also concluded that high beclin-1 was significantly correlated with poor prognostic features. Conversely, other studies concluded that low beclin-1 predominates in TNBC cases and that decreased beclin-1 is associated with poor survival [44–46]. Beclin-1 has dual roles in tumorigenesis. It may supress tumor proliferation and growth by removing defective cellular compounds, or it may exert a cardinal role in cancer initiation and progression by modulating autophagy [9].

The prognostic impact of Bcl-2 varies in accordance with the molecular subtypes of BC [47]. Although the plausible significance of Bcl-2 as a prognostic factor in BC have been previously inspected, our study highlighted its expression in TNBC cases precisely. Bcl-2 was detected in 25 cases (41.67% of the studied cases). Lack of Bcl-2 expression was significantly related to positive nodal metastasis, advanced stage and poor OS. Additionally, negative Bcl-2 was significantly associated with high beclin-1 expression. Our results were consistent with Abdel-Fatah et al. [48] who described that Bcl-2 loss in TNBC was accompanied with doubling the possibility of recurrence as well as death. Similar findings were achieved by others [49–51]. Contrary to our results, Ozretik et al. [52] concluded that elevated Bcl-2 expression is indicative of poor prognosis in TNBC.

Even though Bcl-2 was referred to have a pro-tumourigenic role, consecutive studies reported that its function in different cell types was more sophisticated, and it may be involved in growth inhibition of tumour cells [53]. Bcl-2 is an established anti-apoptotic mediator, however, its role in inhibiting autophagy is becoming more appreciated. Bcl-2 exerts its anti-apoptotic activity via binding to pro-apoptotic proteins containing the Bcl-2 homology 3 (BH3) domain. Bcl-2 is a crucial regulator of autophagy via interacting with beclin-1 at BH3 domain to dampen its pro-autophagic activity. This interaction exerted a leading role in balancing the crosstalk between autophagy and apoptosis [54].

Beclin-1 and Bcl-2 expression levels are basic determinants as to whether apoptosis or autophagy are induced during tumorigenesis and chemotherapy. Bcl‐2 can bind to beclin‐1, via interaction with its BH3 domain, by that preventing autophagy. While, separation of antiapoptotic Bcl‐2 from beclin‐1, subsequently, stimulates autophagy [55].

Hypoxia has been exhibited to enhance autophagy in diverse conditions, and thus autophagy might behave as a survival process for hypoxic cells by means of recycling the cellular component. Pharmacological suppression of autophagy has been demonstrated to induce apoptosis under hypoxic conditions [8]. Moreover, hypoxia-induced autophagy leads to cell survival and resistance to anticancer therapies [56]. It has been determined that BNIP3 and BNIP3L are the downstream targets of HIF-1α-induced autophagy [57].

To our knowledge, this study is the first to address the relation between HIF-1α induced miR-210 overexpression and beclin-1/Bcl-2 complex in TNBC. The current work demonstrated that hypoxic TME within TNBC, as demonstrated by high HIF-1α and high miR-210 mRNA relative expression, was significantly associated with negative Bcl-2 and high beclin-1 expression. Growing evidence stated that some miRNAs could target Bcl-2. Sun et al. [11] studied hypoxia-induced autophagy in colonic carcinoma and reported a significant increase in HIF-1α and miR-210 under hypoxic condition and that decreased miR-210 expression followed inhibition of HIF-1α. They demonstrated that silencing of miR-210 upregulated Bcl-2 expression in colonic carcinoma.

Similarly, Xu et al. [58] investigated miR-210 expression in gastric carcinoma, they reported that miR-210 triggers the oxidative stress enhanced apoptosis in gastric cancer cells possibly by suppressing Bcl-2. A study by Wang et al. [59] reported that upregulation of miR-210 decreased Bcl-2 expression in HTR-8/SVneo trophoblast cell line and miR-210 downregulation increased Bcl-2 expression. Moreover, Chio et al. [60] described that miR-210 could moderate hypoxia-mediated apoptosis of neurons by constraining Bcl-2. Also, Xu et al. [61] demonstrated that miR-210 significantly suppressed Bcl-2 expression in endometriotic cells. They concluded that the hypoxia-mediated miR-210 expression may enhance cell survival and activating autophagy by means of Bcl2/beclin-1 axis. On the contrary, a study by Qiu et al. [62] pointed that miR-210 inhibited neuronal apoptosis and that Bcl-2 expression increased in cells overexpressing miR-210.

Zhang et al. [63] reported that miR-146a endorsed chondrocytes autophagy through inhibiting Bcl-2. Additionally, Wang et al. [64] demonstrated that miR-204 directly targeted Bcl-2. They proclaimed that HIF-1α/miR-204/Bcl-2 pathway led to hypoxia-induced neuronal cells apoptosis.

In the interplay between apoptosis and autophagy, low Bcl-2 levels would indicate that cells are more susceptible to develop apoptosis. Nonetheless, it is notable that the expression levels of additional antiapoptotic proteins, along with Bcl-2 heterodimerization with Bax, are also major determinants to apoptosis susceptibility. Alternatively, as binding of Bcl-2 to beclin-1 prevent autophagy, it is reasonable that low level of Bcl-2 liberates beclin-1 to activate autophagy [65].

Few studies investigating hypoxia-induced autophagy and its relevance to NACT response in TNBC have been published. In this study, high beclin-1, negative Bcl-2 along with hypoxic TME were significantly associated with poor pathologic response. Moreover, HIF-1α as well as miR-210 expression were accurate predictors of treatment response. A study by Sun et al. [11] stated that HIF-1α induced miR-210 triggered autophagy resulting in radio-resistance in colon cancer cells. Wu et al. [66] investigated cisplatin resistance in lung cancer and concluded that HIF-1α enhanced autophagy via increasing the expression of BNIP3 and Beclin-1.

Similarly, Zou et al. [67] described that HIF-1α elevated beclin-1 via activation of the c-Jun pathway and proclaimed that the enhanced autophagy suppresses the radiotherapy-induced reactive O2 species in lung cancer cells. Hypoxia was found to stimulate cytoprotective autophagy and hence diminishes sensitivity of TNBC cells to taxol treatment, proposing that accentuating oxygen tension may be a path to improve chemotherapy sensitivity [68].

There are some limitations in our study, the median survival time cannot be determined as only some cases (25%) have experienced the event by the end of the study (less than half of the studied cases). In addition, the relatively small sample size may also have affected our results.

Long-term follow up of the studied cases is essential to ensure the impact of hypoxia-induced autophagy on patients’ survival. Further studies are mandated to clarify the crosstalk between apoptosis and autophagy. Research on different tumour tissue is necessary to figure out the mechanisms regulating the influence of miR-210 on Bcl-2. A comprehensive insight of different pathways regulating hypoxia-induced autophagy are essential to improve the efficacy of NACT in TNBC.

## Conclusion

High beclin-1 expression and lack of Bcl-2 are significantly associated with hypoxic TME in TNBC. High HIF-1α, miR-210, and beclin-1 expression along with lack of Bcl-2 are significantly associated with poor prognosis as well as poor response to NACT. HIF-1α and miR-210 could accurately predict response to NACT in TNBC.

## Data Availability

The datasets used and/or analyzed during the current study are available from the corresponding author on reasonable request.
